# Foregrounding Dignity and Kindness Across Care Facilities: Aging Around the World

**DOI:** 10.1093/geroni/igaf122.3027

**Published:** 2025-12-31

**Authors:** Luis Cordero, Elissa Kozlov

**Affiliations:** Rutgers School of Public Health, Piscataway, New Jersey, United States; Rutgers School of Public Health, Piscataway, New Jersey, United States

## Abstract

As global demographics shift, innovative models of care are emerging to address the evolving needs and preferences of older adults worldwide. Traditional, one-size-fits-all approaches to long-term care are increasingly insufficient, necessitating adaptable and community-centered solutions. This study draws on field observations from ten countries across South America, Central America, Europe, Asia, and Australia between 2022 and 2023. Site visits encompassed both rural and urban care settings, including nursing homes and community-based programs. A key finding across all settings was the central role of community engagement in enhancing well-being, even within institutional environments. For example, Argentina’s government-funded initiatives, such as Estaciones Saludables and free day centers, extend opportunities for older adults to remain engaged in community life and delay institutionalization. Similarly, intergenerational exchange programs within care settings foster mutual benefits for residents and the broader community. Additional innovative models include Spain’s integration of coworking spaces within nursing homes, Australia’s Community Home Model, and Nepal’s extensive community event programs for older adults. These approaches highlight the importance of fostering social connections and reimagining traditional care structures to align with cultural contexts and demographic changes. As societies worldwide adapt to aging populations, the future of long-term care lies in models that emphasize community integration, social engagement, and cultural responsiveness.

